# Simulation of the water-based hybrid nanofluids flow through a porous cavity for the applications of the heat transfer

**DOI:** 10.1038/s41598-023-33650-w

**Published:** 2023-04-28

**Authors:** Taza Gul, Saleem Nasir, Abdallah S. Berrouk, Zehba Raizah, Wajdi Alghamdi, Ishtiaq Ali, Abdul Bariq

**Affiliations:** 1 DoST, Directorate General of Science & Technology, Khyber Pakhtunkhwa, Peshawar, 25000 Pakistan; 2 Mechanical Engineering Department, Khalifa University of Science and Technology, P. O. Box 127788, Abu Dhabi, United Arab Emirates; 3 Center for Catalysis and Separation (CeCas), Khalifa University of Science and Technology, P.O. Box 127788, Abu Dhabi, United Arab Emirates; 4 Department of Mathematics, College of Science, Abha, King Khalid University, Abha, Saudi Arabia; 5 Department of Information Technology, Faculty of Computing and Information Technology, King Abdulaziz University, 80261 Jeddah, Saudi Arabia; 6 Department of Mathematics and Statistics College of Science, King Faisal University, P. O. Box 400, 31982 Al-Ahsa, Saudi Arabia; 7 Department of Mathematics, Laghman University, Mehterlam, Laghman, 2701 Afghanistan

**Keywords:** Mathematics and computing, Physics

## Abstract

This study looks at the natural convections of Cu + Al_2_O_3_/H_2_O nanofluid into a permeable chamber. The magnetic field is also executed on the flow field and the analysis has been approached numerically by the control volume method. The study of hybrid nanofluid heat in terms of the transfer flux was supplemented with a wide range of parameters of hybrid nanofluid fractions, Rayleigh numbers Hartmann numbers and porosity factor. It's also determined that the flow and thermal distribution are heavily affected by the concentration of the nanoparticles. The concentration of nanoparticles increases the transport of convective energy inside the enclosure. The primary findings demonstrate that a rise in both the Rayleigh number and Darcy number leads to an improvement in convective heat transfer within the enclosure. However, the porosity has a negligible effect. Additionally, the rotation in a clockwise direction has a beneficial impact on the dispersion of heat transfer throughout the cavity. Furthermore, it is concluded that hybrid nanofluids are more reliable than conventional fluids in improving thermal properties.

## Introduction

Mutual convectional motion and energy transmission have been inspected in an enormous quantity of investigations for a centuries due to its technical uses in technogolical scientific sectors. The new addition of hybrid nanofluids has diminished a more important role in improving the thermal behavior of fluids for energy resources. The flow of nanofluid within an enclosed chamber is managed by Khanafer et al.^1^ Using the finite volume technique. The nanofluid consists of a copper nanofluid stream in an enclosed chamber studied by Haq et al.^2^. The CNTs nanofluid flow in a wedge-shaped cavity for thermal applications has been investigated by^3^. The impact of the volumetric fraction on convective analysis was examined by Khan et al.^4^ considered a right-angled enclosure for the enhancement of the thermal field. Closed and porous chambers are mostly used in the modern sciences and have many applications including solar energy systems, electronic cooling, and gas sensing devices. The shapes of these cavities are of different shapes according to scientific requirements and the main purpose of these chambers is the improvement of cooling of the thermal devices. At some point, these cavities are also used for distillation. The potential growth of heat transfer to the hollow can be found by an outside cold air stream. An extensive number of available literature addresses the features of flux, heat transfer, distillation, design problems, etc. Many researchers^5–7^ have investigated several physical circumstances, which can be divided are more prominent to fulfill the energy crises that are mostly required for advanced technologies. The key role of the researchers is to obtain these energies based on some conditions including cheap resources and environmentally friendly. The hybrid nanofluids consisting Cu and Al2O_3_ nanomaterials are displayed in^8–10^ for the enhancement of heat transfer using varities of mathematical models. herefore, most of the researchers used nanomaterials or nanoparticles to prepare the nanofluids for the different kinds of chambers^11–13^. Some of the researchers concentrated on porous cavities on the use of the Darcy theory as seen in^14–17^. Kumar^18^, Selimefendigil et al.^19^ Studied the free convection in a rippling cavity occupied with a permeable medium.

The combination of two dissimilar dispersal nanoparticles in the same base fluid having different chemical properties carried out hybrid nanofluids. Researchers have focused on these types of nanofluids because of their advanced thermophysical performance. Al-Kouz et al.^20^ Used a hybrid nanofluid for thermal applications with the entropy generation combination. They observed that these nanocomposites are much more efficient in enhancing the thermal behavior of the base liquid. Salawu et al.^21^ Used nonlinear analysis taking into consideration, hybrid nanofluids with magnetic properties. They noted that their results, optimize energy resources. Rahman et al.^22^ Used the adverse flow of the hybrid nanofluid taking into account the surface reduction. Jahan et al.^23^ Investigated the flow of hybrid nanofluids on a moving needle surface. Khan et al.^24^ looked at micropolar nanofluids for thermal applications. Bilal et al.^25^ studied hybrid nanofluid flow through porous space using the sliding surface of a tube. Gul et al.^26,27^ reviewed nanofluid flux analysis to improve the thermal performance of the base solvent. Arin et al.^28^ Examined the nanofluid flux employing heat and mass transfer analysis. Zeeshan et al.^29,30^ looked at nanofluid flux for thermal and biotechnological applications.

Thermal transport of hybrid nanofluids is carried out by^31^. They have considered different inclinations. Rudraiah et al.^32^ We're the pioneer to introduce the flow phenomena considering a closed chamber. The nano-fluidic performance through the impact of fusion energy transmission have been investigated by^33–35^. They also employed the famous fluid modle known Buongiorno for unstable flux and energy transmission of nanofluids. A variety of publications have been published on the improvement of nanofluid heat transfer over the past few years^36–40^.

This analysis aims to simulate the impact of heat radiations on the performance of hybrid nanofluids in the presence of magnetic forces via CVFEM approach. The roles of the permeable parameter, Magnetic and nanoparticle volume fraction are presented as outputs. In the framework of the above conversation, the objective of the study is to investigate the flow of hybrid nanofluids in a porous cavity for thermal applications. Cu and Al_2_O_3_ are used as nanoparticles and hybrid nanofluids have proven to be more important in enhancing the heat transfer mechanism. This study utilizes experimental correlations to examine the thermal and physical properties of nanofluids, such as thermal conductivity and dynamic viscosity.

### Description of the problem

Cu and Al_2_O_3_ are used in water for the production of hybrid nanofluids. Hybrid nanofluid is considered in a porous chamber where there is a magnetized field. The magnetic field was applied in the proper direction at a 90^0^ angle. The space is considered porous in the flow field of the hybrid nanofluid. The construction of the sinusoidal wall is defined as.

1 $$b = a \cdot \left( {1 - \varepsilon } \right)^{2}$$

The fundamental model expression are as follows by Boussinesq-Darcy and the nonequilibrium thermal model with the temperature model^32,33^,

2 $$\nabla \cdot \mathop V\limits^{ \to } = 0,$$

3 $$\rho_{hnf} \beta_{hnf} \vec{g}\left( {\tilde{T}_{hnf} - \tilde{T}_{c} } \right) \wedge + \frac{{\mu_{hnf} }}{K} + \nabla p + \sigma_{hnf} \left( {\vec{V} \times \vec{B}} \right) \wedge = 0,$$

4 $$\frac{{h_{hnfs} }}{{\rho_{s} \left( {cp} \right)_{s} \left( {1 - \varepsilon } \right)}}\left( {\tilde{T}_{hnf} - \tilde{T}_{s} } \right) + \frac{{k_{s} }}{{\rho_{s} \left( {cp} \right)_{s} }}\nabla^{2} \tilde{T}_{s} = 0,$$

where the characteristics of hybrid nanofluids are^8^,

5 $$\begin{aligned} \phi & = \phi_{Cu} + \phi_{{Al_{2} O_{3} }} ,\,\,\,\, \\ \rho_{hnf} & = \left( {1 - \phi } \right)\rho_{f} + \rho_{{Al_{2} O_{3} }} \phi_{{Al_{2} O_{3} }} + \rho_{Cu} \phi_{Cu} , \\ \,\left( {\rho cp} \right)_{hnf} & = \left( {1 - \phi } \right)\left( {\rho cp} \right)_{f} + \left( {\rho cp} \right)_{{Al_{2} O_{3} }} \phi_{{Al_{2} O_{3} }} + \left( {\rho cp} \right)_{Cu} \phi_{Cu} , \\ \left( {\rho \beta } \right)_{hnf} & = \left( {1 - \phi } \right)\left( {\rho \beta } \right)_{f} + \left( {\rho \beta } \right)_{{Al_{2} O_{3} }} \phi_{{Al_{2} O_{3} }} + \left( {\rho \beta } \right)_{Cu} \phi_{Cu} . \\ \end{aligned}$$

6 $$\sigma_{hnf} = \sigma_{f} + 3\frac{{\left( {\frac{{\sigma_{np} }}{{\sigma_{f} }} - 1} \right)\phi }}{{\left( {1 - \frac{{\sigma_{np} }}{{\sigma_{f} }}} \right)\phi + \left( {\frac{{\sigma_{np} }}{{\sigma_{f} }} + 2} \right)}}\sigma_{f}$$

The $k_{nf} ,\,\,and\,\,\mu_{nf}$ stated by the KKL (Koo-Kleinstreuer-Li) model as^33^,

7 $$\mu_{hnf} = \mu_{f} \left( {\frac{1}{{\left( {1 - \phi_{Cu} - \phi_{{Al_{2} O_{3} }} } \right)^{5/2} }} + \frac{{k_{Brownian} }}{{\Pr \,\,k_{f} }}} \right)$$

8 $$k_{hnf} = k_{f} - 3k_{f} \frac{{\left( {k_{f} - k_{np} } \right)\phi }}{{\left( {k_{f} - k_{np} } \right)\phi + \left( {k_{np} + 2k_{f} } \right)}} + 5 \times 10^{4} \rho_{f} \phi \left( {\frac{{k_{b} \tilde{T}}}{{\left( {\rho d} \right)_{np} }}} \right)^{1/2} c_{p,f} g^{\prime}\left( {\tilde{T},d_{p} ,\phi } \right)$$

where $g^{\prime}\left( {\tilde{T},\,\,d_{p} ,\,\,\phi } \right)$ function stated as:

9 $$\begin{aligned} g^{\prime}\left( {\tilde{T},d_{p} ,\phi } \right) & = \ln \left( {\tilde{T}} \right)\left( \begin{gathered} b_{1} + b_{2} \ln \left( {d_{p} } \right) + b_{3} \ln \left( \phi \right) + b_{4} \ln \left( {d_{p} } \right) + b_{5} \ln \left( {d_{p} } \right)^{2} + \hfill \\ b_{6} + b_{7} \ln \left( {d_{p} } \right) + b_{8} \ln \left( \phi \right) + b_{9} \ln \left( \phi \right)\ln \left( {d_{p} } \right) + b_{10} \ln \left( {d_{p} } \right)^{2} \hfill \\ \end{gathered} \right) \\ R_{f} & = \frac{{d_{p} }}{{k_{p,eff} }} - \frac{{d_{p} }}{{k_{p} }} = 4 \times 10^{ - 8} km^{2} /W \\ \end{aligned}$$

Also, $b_{i} ,\,\,\,\,i = [0,\,\,\,10]$ is depending on the nanoparticles type.

The dimensionless variables are:

10 $$v = - \frac{\partial \psi }{{\partial x}},\,\,u = \frac{\partial \psi }{{\partial y}},\,\Psi = \frac{\psi }{{\alpha_{nf} }},\,\left( {X,Y} \right) = \frac{{\left( {x,y} \right)}}{l},\theta_{s} = \frac{{\left( {\tilde{T}_{s} - \tilde{T}_{c} } \right)}}{{\left( {\tilde{T}_{h} - \tilde{T}_{c} } \right)}},\,\theta_{nf} = \frac{{\left( {\tilde{T}_{nf} - \tilde{T}_{c} } \right)}}{{\left( {\tilde{T}_{h} - \tilde{T}_{c} } \right)}}.$$

The model expression (9) in (2–4) gives the following non-designable sets of differential equations:

11 $$\begin{aligned} \frac{{\partial^{2} \Psi }}{{\partial X^{2} }} + \frac{{\partial^{2} \Psi }}{{\partial Y^{2} }} & = - \frac{{L_{6} }}{{L_{5} }}Ha\left( {\frac{{\partial^{2} \Psi }}{{\partial X^{2} }}\cos^{2} \gamma + 2\frac{{\partial^{2} \Psi }}{\partial X\partial Y}\cos \gamma \sin \gamma + \frac{{\partial^{2} \Psi }}{{\partial Y^{2} }}\sin^{2} \gamma } \right)\, \\ & \quad - \frac{{L_{3} }}{{L_{4} }}\frac{{L_{2} }}{{L_{5} }}Ra\frac{{\partial \theta_{nf} }}{\partial X} - \frac{{L_{5} }}{{L_{1} }}\frac{\Pr }{{Da}}\frac{\partial \Psi }{{\partial X}}, \\ \end{aligned}$$

12 $$\frac{{\partial^{2} \theta_{nf} }}{{\partial X^{2} }} + \frac{{\partial^{2} \theta_{nf} }}{{\partial Y^{2} }} = \frac{{\partial \theta_{nf} }}{\varepsilon \partial X}\frac{\partial \Psi }{{\partial Y}} - \frac{{Nhs\left( {\theta_{s} - \theta_{nf} } \right)}}{\varepsilon } - \frac{{\partial \theta_{nf} }}{\varepsilon \partial Y}\frac{\partial \Psi }{{\partial X}}$$

13 $$\frac{{\partial^{2} \theta_{s} }}{{\partial X^{2} }} + \frac{{\partial^{2} \theta_{s} }}{{\partial Y^{2} }} = - \frac{{Nhs\left( {\theta_{nf} - \theta_{s} } \right)}}{\varepsilon }$$

where

14 $$\begin{aligned} L_{1} & = \frac{{\rho_{hnf} }}{{\rho_{f} }},\,\,\,L_{2} = \frac{{\rho_{hnf} \left( {cp} \right)_{hnf} }}{{\rho_{f} \left( {cp} \right)_{f} }},\,\,\,L_{3} = \frac{{\rho_{hnf} \left( \beta \right)_{hnf} }}{{\rho_{f} \left( \beta \right)_{f} }},\,\,L_{4} = \frac{{k_{hnf} }}{{k_{f} }},L_{5} = \frac{{\mu_{hnf} }}{{\mu_{f} }},\,\,\,\,L_{6} = \frac{{\sigma_{hnf} }}{{\sigma_{f} }},\,\,\,\, \\ Ra & = \frac{{gK\rho_{f} \left( \beta \right)_{f} \Delta \tilde{T}}}{{\mu_{f} \alpha_{f} }},\,\,Nhs = \frac{{h_{hnfs} l^{2} }}{{k_{hnf} }},\,\,\,\delta_{s} = \frac{{k_{hnf} }}{{k_{f} \left( {1 - \varepsilon } \right)}},\,\,\,\,\,Ha = \frac{{KB_{0}^{2} \sigma_{f} }}{{\mu_{f} }},Da = \frac{k}{{l^{2} }} \\ \end{aligned}$$

Given that the inner side is assumed to be heated, the boundaries conditions are as follows:

15 $$\left. \begin{gathered} {\text{On}}\,\,{\text{all}}\,\,{\text{walls}} \to \Psi = 0, \hfill \\ {\text{On}}\,\,{\text{the}}\,\,{\text{outer}}\,\,\,{\text{wall}} \to \theta_{s} = 0,\,\,\,\theta_{nf} = 0, \hfill \\ {\text{On}}\,\,{\text{the}}\,\,{\text{inner}}\,\,{\text{wall}} \to \theta_{s} = 1,\,\,\,\,\theta_{nf} = 1. \hfill \\ \end{gathered} \right\}$$

In case, when the walls is cool, then:

16 $$Nu_{loc} = \frac{{k_{nf} }}{{k_{f} }}\frac{{\partial \theta_{nf} }}{\partial r},\,\,\,\,\,Nu_{ave} = 0.5\pi \int\limits_{0}^{2\pi } {Nu_{loc} dr}$$

## Testing and checking the grid's independence

The modelled momentum and energy problems should consequently be numerically investigated. To handle the leading model systems of equations subject to the associated boundary constraints computationally, control volume finite element method has been used (CVFEM). Such approach combines the finite volume with finite element techniques^6^. The Matlab software platform was used to create the programming code that was used to execute all computational modeling. By comparing the numerical output obtained by the current code with the previous approaches, confirmation of calculated results is accomplished in order to confirm the accuracy of the current physical description. Consequently, the reliability of the current code has been extensively tested in comparison to the studies^33^. The hemispheric natural convection cavity packed with nanofluid has been investigated in this research using the grid independence test.Ra = 50, Ha = 10, and Da = 20 are the specified settings for the physical parameters that will be used to test the grid size. This test has been run for two distinct mesh size scenarios.

**Figure 1:**
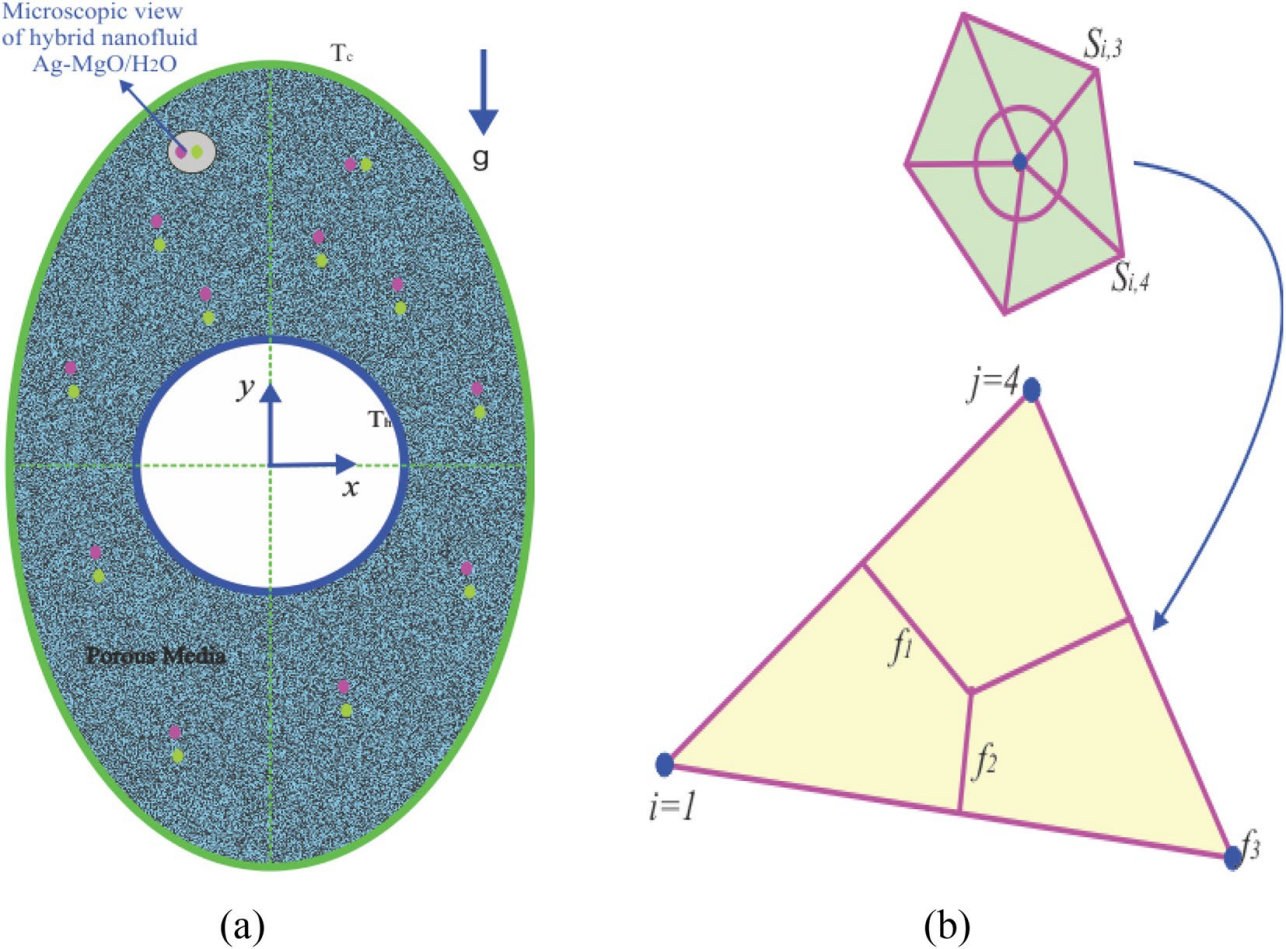
(**a**) Geometrical configuration and imposed boundary constraints using (**b**) A sampler triangular components and its associated volume control.

**Figure 2:**
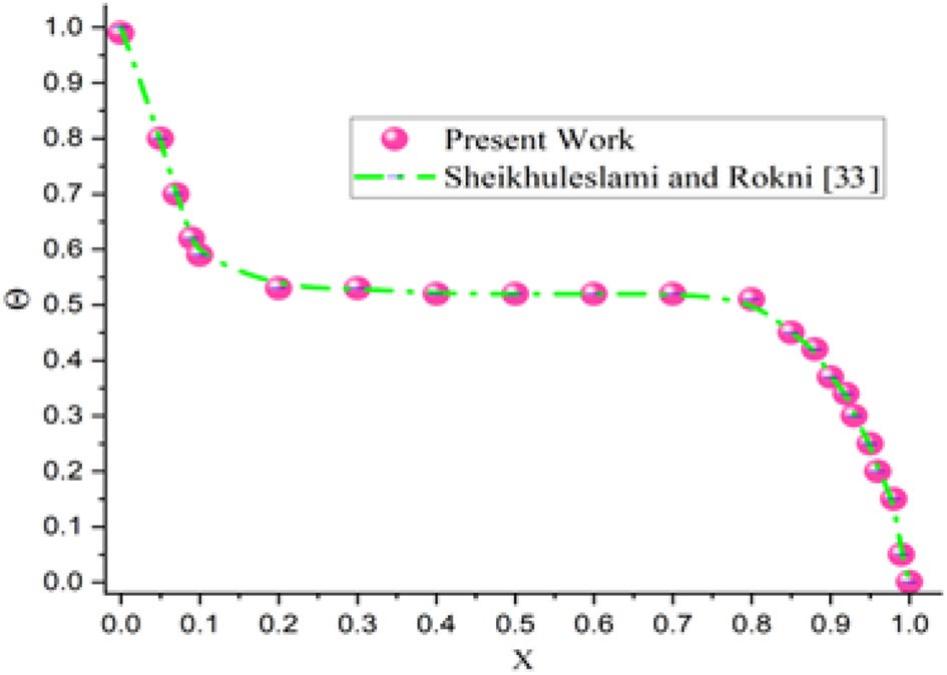
Verification of current outcomes with previous work^38^.

**Figure 3:**
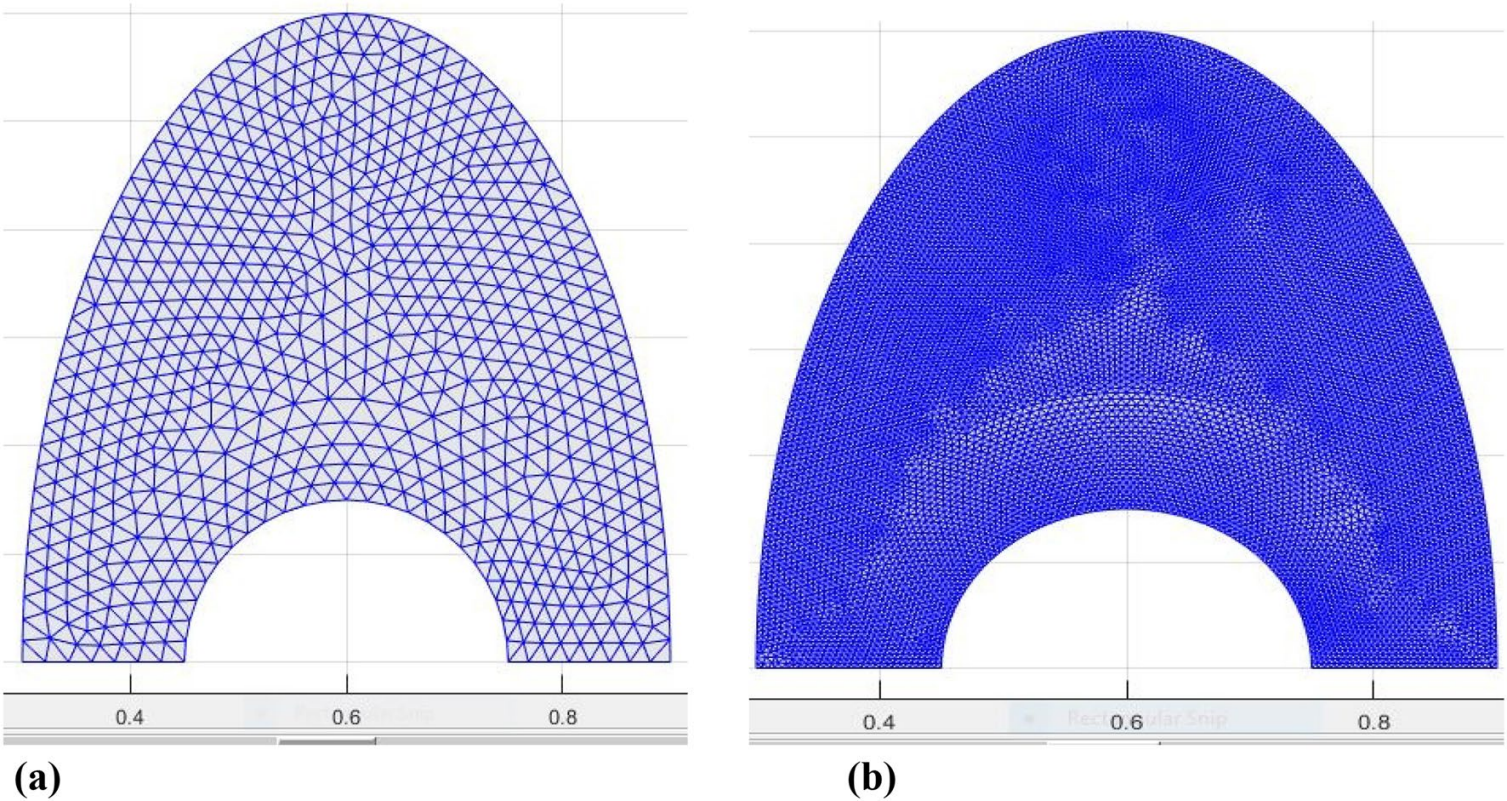
The grid presentation of the proposed model.

**Figure 4:**
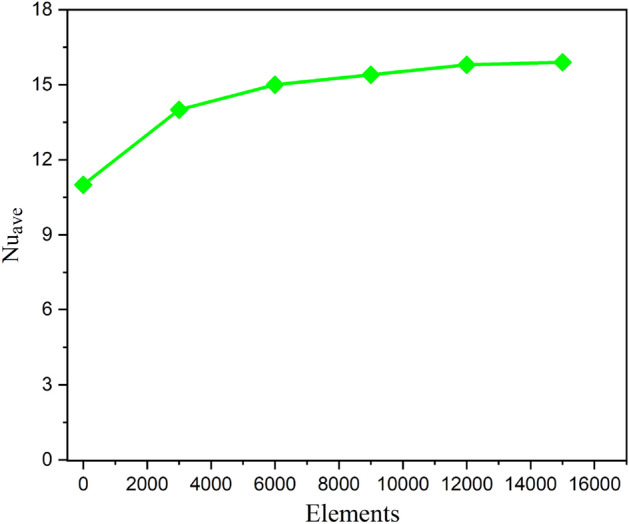
The grid test profile.

The geometric configuration and boundary assumption using are displayed in Fig. 1a and a triangular element of the sampler and the associated volume control are shown in Fig. 1b. The obtained rerults validation is shown in Fig. 2. The grid presentation of the proposed model as shown in Fig. 3. Therefore, 15,920 elements are decided for this mathematical calculation to satisfy the criteria of the grid sensitivity test, as shown in Fig. 4.

## Findings and discussion

The computational results for the temperature distributions and natural convection nanofluid flow patterns in a porous enclosure are presented in this section of the paper. This investigation focused on the important dimensionless parameters Ra, Da, Ha, and volume fraction of nanofluid. For the relevant range of examined parameters, such as Ra = 50, Da = 5 and Ha = 5, numerical simulations have been performed. To view high grids, the computational volume finite element (CVFEM) technique was applied. According to the published research^8–10^, Table 1 displays the thermo—physical characteristics of the base fluid, Cu, and Al2O3 nanoparticles. Table 2 lists recent findings that have been supported by literature. The published work^32,33^ and the current results have been found to be in excellent correlation. Cu + Al_2_O_3_.

**Table 1:** The numerical properties of Cu & Al_2_O_3_ nanoparticles as^8–10^.

Properties	Water	Cu	Al_2_O_3_
Density $\left( {\rho = {\text{kg}}/{\text{m}}^{3} } \right)$	997.10	8933	3970
Heat capacity $\left( {C_{p} = j/kgk} \right)$	04,179	00,385	765
Thermal conductivity $\left( {k = {\text{W}}/{\text{m}}\;{\text{k}}} \right)$	0.6130	00,401	40
Thermal expansion $\left( {\beta \times 10^{5} = {\text{K}}^{ - 1} } \right)$	00,021	$1.67 \times 10^{ - 5}$	$0.85 \times 10^{ - 5}$
Electrical conductivity $\left( {\sigma = \frac{s}{m}} \right)$	$5.5 \times 10^{ - 6}$	$5.96 \times 10^{ - 7}$	$1 \times 10^{ - 10}$

**Table 2:** Comparison of current results and published work on traditional fluids having common parameters**.**

$Ha$	$Nu_{ave}$ ^32^	$Nu_{ave}$ ^33^	$Nu_{ave}$[Present]
0	2.35261	2.3738	2.38218
5	2.17632	2.19421	2.199210
10	2.0347	2.050321	2.062180
15	1.5564	1.596542	1.618743

**Figure 5:**
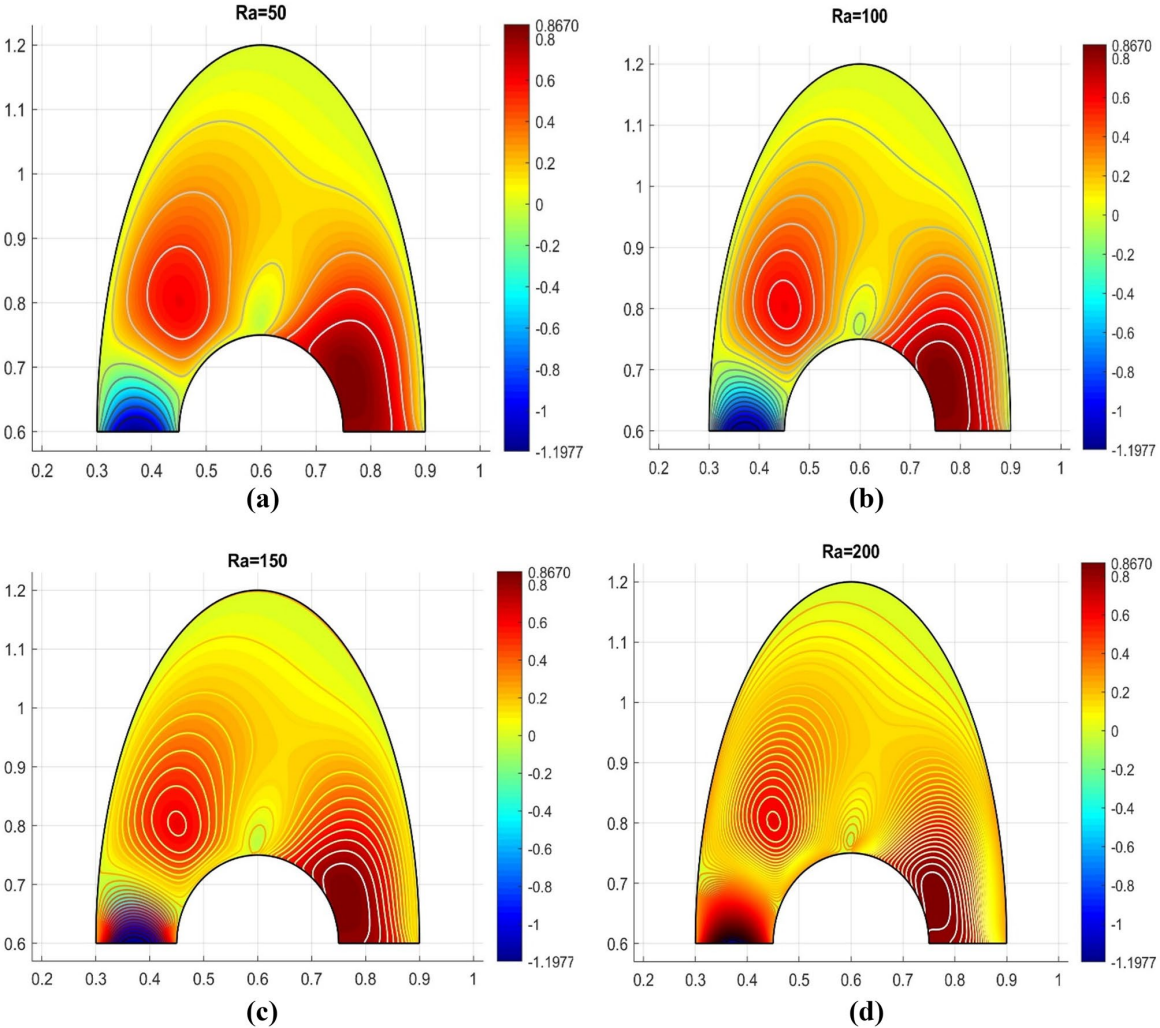
Various Rayleigh numbers for the velocity profile.

For different Rayleigh numbers (Ra = 50, 100, 150, 200), Fig. 5a–d displays the outflows within the investigated cavity filled with Cu + Al_2_O_3_/H_2_O anofluid. It should be observed that the streamlining created two contours on front of the cavity's warmed surface and one contour next to its cooler surface. The warmed streamlining contours exceed the cooler single contour with decreasing values of the Ra. A higher Ra causes the cooling end to dominate.It can be the result of convectional transportation occurring within the cavity. It demonstrates how heat in the cavity fluid causes a drop in densities. In other words the fluid's flow rate is determined by Rayleigh's number, which is connected to movement via buoyancy, also known as free convection. Convectional stram is negligible and the conducting state is steady when the magnitude of Ra is low.

**Figure 6:**
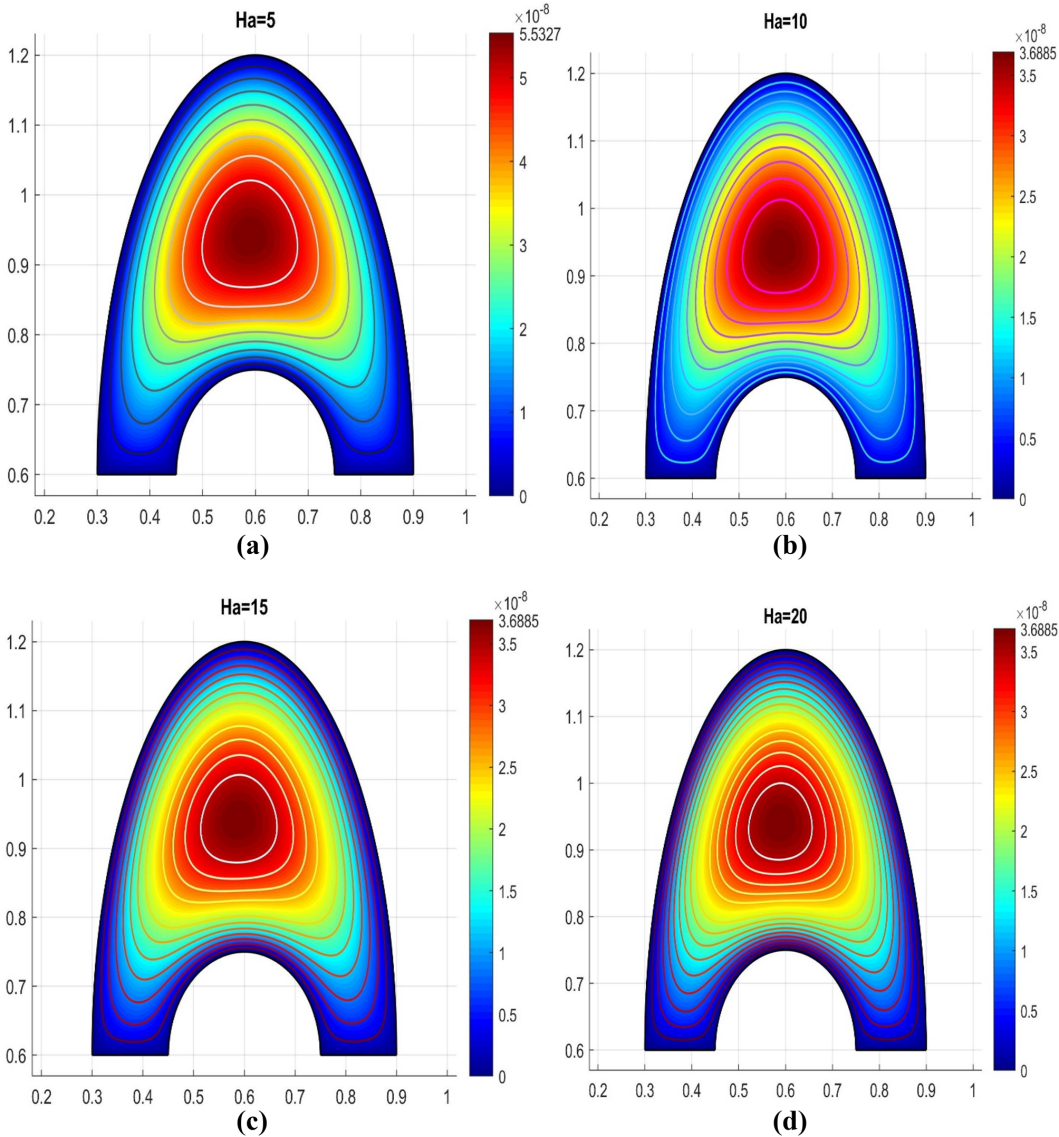
Various Hartmann numbers for the velocity profile.

The Hartmann number Ha can be used to characterize the forces produced by electromagnetism interactions with viscosity changes caused by thermal changes. Figure 6a–d demonstrates the streamlineings within the investigated cavities that were exposed to the transversal uniform magnetic field (Ha = 5, 10, 15, 20) and had thermal variations on each edge. The streamlines developed two smaller contours in the hot surface and one major contour close the coldest surface when there was no magnetic field influence (Ha = 0). The intensity of such contours moved further toward the cavity's bottom for larger Hartmann numbers as a result of the flow's increasing resistance. The fluid flowing through the cavity is magnetized constricted, which slows down the temperature transfer between the two surface. Higher levels of Ha cause the streamlining to begin spreading away from the surface.

**Figure 7:**
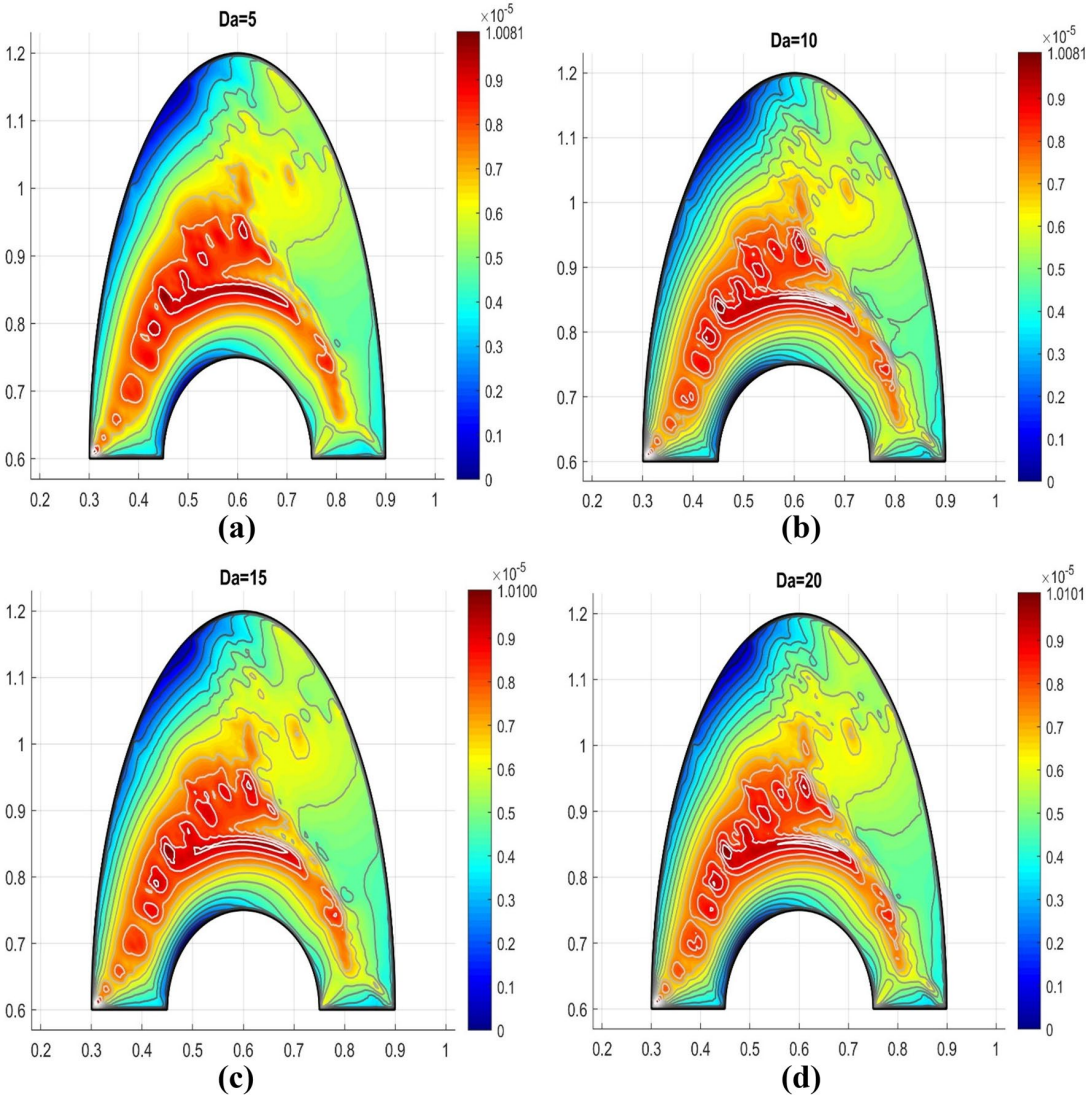
Variable porosity parameter for the porosity factor.

Around the cavity's porous structure, the significance of the Darcy number gets considerable. The transparency of the media rises as Darcy's number improves, permitting the nanofluid movement into it. For greater values of the Darcy number Da, it was portrayed by the streamlining aggregation on each side of a permeable medium and flowing into it all. The cooler surface has a stronger pattern comparing to the warmer portion; this may be because cooler liquid has a lower penetration. According to the observations, Fig. 7a–d clearly indicates that at higher values of the Da = 5, 10, 15, 20, the heat near the porous medium and center region of the cavity displays softer streamlines.

**Figure 8:**
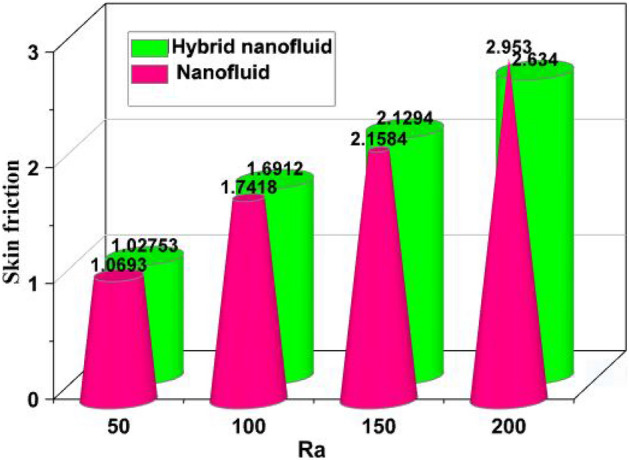
Skin friction versus Ra.

**Figure 9:**
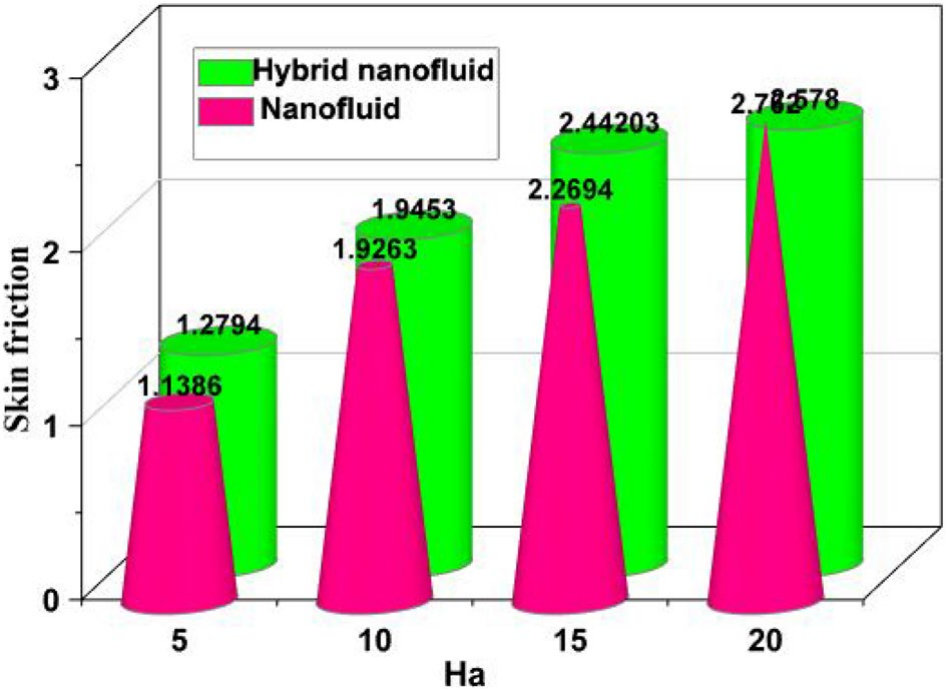
Skin friction versus Ha.

**Figure 10:**
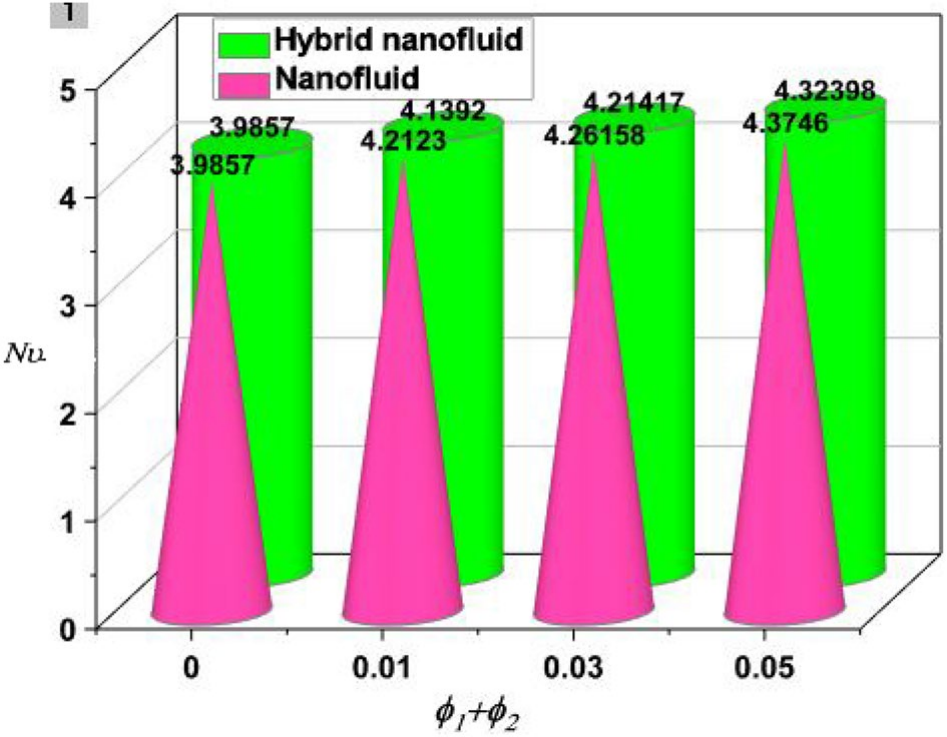
Nusselt number Vs volume fraction.

**Figure 11:**
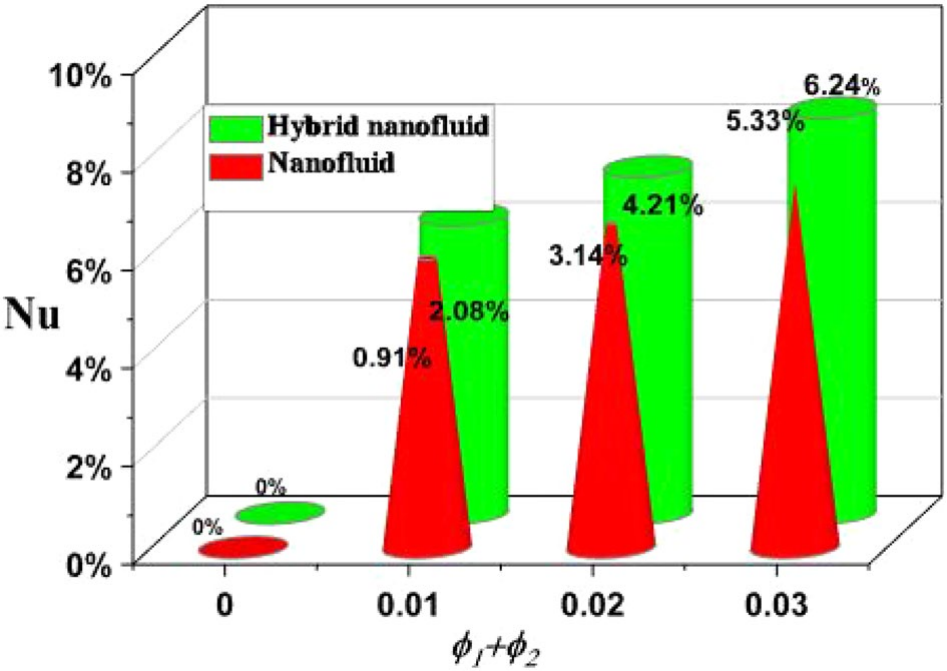
Percent improvement of heat transfer.

By taking the case of the hybrid nanofluid, as depicted in Fig. 8, the Ra has a stronger impact on drag force due to its higher values. Comparable to how growing Ha enhance drag force, Fig. 9 illustrates that phenomenon. The rate of heat transfer increases with increasing fractional volumes of nanoparticles, as shown in Fig. 10. According to Fig. 11, hybrid nanofluids are more successful in enhancing heat transmission due to the rise in the volumetric proportion of nanostructures.The percentage wise improvement demonstrats that hybrid nanofluids are extra consistent in improving the rate of energy transmission. Current results are compared and validated against available literature, as shown in Table 2.

## Conclusion

The natural convective energy transmission of a nanoparticles inside of a sami circular cavity has been considered as a novel geometry in this research. By the use of CVFEM approach combination with magnetic model was used to carry out the computational modeling for the Al_2_O_3_ + Cu/H_2_O nanofluids. The findings of this analysis for the relevant nondimensional factors Ra, Ha, Da and volume fraction of nanofluid were reported. We have quantitatively investigated the variations of streamlines, local, and average Nusselt numbers for the mentioned physical factors. The following is a summary of the research's detailed analyses:

- From the explanations of fluid (Cu + Al_2_O_3_/H_2_O) motion decreases considerably around the channel's middle as a result of a strengthening the magnitude of $Ra,Ha$ and $\phi$.
- The liquid within the container was driven toward the cavity's upper surface for larger values of Ra.
- In comparison to traditional fluids, the hybrid nanofluid's temperature profile appears to be consistently higher.
- For stronger Ha, the magnitude of skin friction is enhanced.
- As the magnitude of $\phi$ is increased, the local Nusselt number rises.

In order to explore the heat transfer performance of hybrid nanofluids in a semi-circular cavity, some potential future work that could be undertaken include, conducting a comparison study between the heat transfer performance of the various hybrid nanofluid and conventional heat transfer fluids in the semi-circular cavity to determine the efficiency and effectiveness of the hybrid nanofluid. Future work on hybrid nanofluid heat transfer in a semi-circular cavity can help to improve our understanding of the heat transfer performance of these fluids and their potential applications in various industries.

## Data Availability

The datasets used and/or analysed during the current study available from the corresponding author on reasonable request.

## List of symbols

$u,v$: Elements of velocity (m s^−^^1^)
$B_{0}$: Strength of magnetic field (N m A^−^^1^)
$f$: Dimensional velocity field
$T$: Temperature of fluid (K)
$T_{l} ,T_{u}$: Temperature of Lower, Upper wall (K)
Ra: Rayleigh number
Ha: Hartmann number
$$K$$: Permeability
$$H$$: Hartmann numbers

## Abbreviations

KKL: Koo–Kleinstreuer–Li
CVFEM: Control volume finite element method
CNTs: Carbon nanotubes

## Greek symbols

$\mu$: Dynamic viscosity (mPa)
$\theta$: Dimensional thermal field
$$\gamma$$: Inclination
$\rho_{f}$: Density of base fluid (kg m^−^^3^)
$\sigma_{nf}$: Nanofluid electrical conductivity
$\phi$: Volume fraction of nanoparticle
$$\psi$$: Stream function
$\varepsilon$: Porosity of porous medium

## Subscripts

$$hnf$$: Hybrid nanofluid
$$nf$$: Nanofluid
$$f$$: Base fluid
$${\text{Al}}_{2} {\text{O}}_{3}$$: Alumina
$${\text{Cu}}$$: Coper
